# Exploring the Impact of Microgravity on Gene Expression: Dysregulated Pathways and Candidate Repurposed Drugs

**DOI:** 10.3390/ijms26031287

**Published:** 2025-02-02

**Authors:** Karina Galčenko, Marilena M. Bourdakou, George M. Spyrou

**Affiliations:** Bioinformatics Department, The Cyprus Institute of Neurology and Genetics, 2371 Nicosia, Cyprus; qee.galcenko@gmail.com (K.G.); marilenab@cing.ac.cy (M.M.B.)

**Keywords:** microgravity, spaceflight, gene expression, transcriptomics, drug repurposing, space health

## Abstract

Space exploration has progressed from contemporary discoveries to current endeavors, such as space tourism and Mars missions. As human activity in space accelerates, understanding the physiological effects of microgravity on the human body is becoming increasingly critical. This study analyzes transcriptomic data from human cell lines exposed to microgravity, investigates its effects on gene expression, and identifies potential therapeutic interventions for health challenges posed by spaceflight. Our analysis identified five under-expressed genes (*DNPH1*, *EXOSC5*, *L3MBTL2*, *LGALS3BP*, *SPRYD4*) and six over-expressed genes (*CSGALNACT2*, *CSNK2A2*, *HIPK1*, *MBNL2*, *PHF21A*, *RAP1A*), all of which exhibited distinct expression patterns in response to microgravity. Enrichment analysis highlighted significant biological functions influenced by these conditions, while in silico drug repurposing identified potential modulators that could counteract these changes. This study introduces a novel approach to addressing health challenges during space missions by repurposing existing drugs and identifies specific genes and pathways as potential biomarkers for microgravity effects on human health. Our findings represent the first systematic effort to repurpose drugs for spaceflight, establishing a foundation for the development of targeted therapies for astronauts. Future research should aim to validate these findings in authentic space environments and explore broader biological impacts.

## 1. Introduction

Understanding the effects of space travel on human health is essential, as space missions pose unique risks and challenges. This knowledge is critical not only for safeguarding astronauts but also for its broader implications, offering insights into conditions such as aging, osteoporosis, and cardiovascular diseases. The environmental stressors encountered in space—particularly radiation and microgravity—can trigger various health issues, potentially intensifying as the mission duration increases. Addressing these factors is crucial to enabling safer long-term space exploration and advancing our understanding of human physiology.

NASA identifies five main risks in spaceflight: microgravity, radiation, isolation and confinement, hostile environments, and distance from Earth [1,2]. Among these, microgravity presents substantial physiological challenges, impacting the body across three primary phases: initial adaptation, prolonged exposure, and readaptation to Earth’s gravity [3]. Key physiological changes include fluid redistribution, plasma volume reduction, and decreased hemoglobin levels, collectively leading to a condition termed space anemia [4]. Microgravity also adversely affects cardiovascular health, bone density, and muscle mass, with fluid shifts contributing to sensory changes, such as altered olfaction [5]. Additionally, structural changes in the brain’s gray matter have been observed, underscoring the extensive neurological impact of microgravity [6,7].

Space radiation, though not the primary focus, is another critical concern, damaging DNA and increasing the risk of carcinogenesis [8]. Astronauts face chronic radiation exposure from galactic cosmic rays (GCRs), which differ significantly from Earth’s radiation, causing oxidative damage and genetic mutations [9,10,11]. NASA has increased the career radiation dose limit to 600 mSV, a threshold that missions to Mars are projected to exceed substantially [9,12].

The isolation and confinement of space, combined with the lack of a circadian rhythm, present risks to mental health and sleep, potentially affecting mission performance [1]. Environmental conditions during spaceflight must be closely controlled to mimic Earth, but challenges such as communication delays and limited resources heighten stress and suppress immune function, further complicating long-term space missions [1,3,5,13,14].

Space travel presents unique health challenges, making personalized strategies essential, especially as astronaut diversity grows. The NASA Twin Study demonstrated long-lasting physiological changes, emphasizing the need for tailored approaches [13]. Bioinformatics and omics technologies—genomics, proteomics, and metabolomics—enable the development of unique health profiles and personalized medical plans for astronauts, which are critical due to limited crew numbers and resource constraints [15]. Genomics provides insight into disease predispositions but lacks adaptability to changing conditions. Transcriptomics, in contrast, captures real-time gene expression changes in response to space-related stressors, aiding in the development of personalized space travel approaches. This research focuses on microarray technology, which, though limited to known genes, is cost-effective and well suited to analyzing gene expression changes due to spaceflight [16]. Identifying differentially expressed genes between ground-based controls and space samples can uncover the molecular mechanisms underlying astronauts’ adaptation to space, which is critical for long-term space missions and personalized medicine advancements.

Understanding these mechanisms is key to developing effective countermeasures. Space-related factors disrupt the immune system, increasing susceptibility to infections. While current strategies are generalized, precision medicine offers a means of delivering individualized immune countermeasures by targeting the specific molecular changes induced by space conditions [17].

Numerous studies have analyzed astronauts’ transcriptomic data to identify differentially expressed genes (DEGs) between pre-flight and during-flight states, focusing on changes in gene expression and protein function due to space conditions. Sakharkar and Yang [18] examined gene expression changes in astronauts’ hair follicles across different stages of spaceflight using microarray data, identifying the over-expressed genes *LIMCH1* and *IER3*, which are associated with cancer growth and progression, and the under-expressed genes *NFATC1*, linked to osteoporosis, and *KIDINS220*, which potentially leads to neurological and cardiovascular issues. Hwang et al. [19] analyzed cardiac progenitor spheres exposed to long-term microgravity using RNA-seq data, and found the over-expression of gene *CCBE1*, which is crucial for heart development, and the under-expression of gene *CD24*, which is a marker for pluripotent stem cells. This research expands on these findings by comparing DEGs from various datasets under microgravity conditions to identify novel molecular targets for therapeutic intervention.

Given the limited opportunities for direct human space travel, collecting comprehensive and continuous astronaut data presents a significant challenge. Thus, human cell lines are utilized to compare molecular responses to existing knowledge, providing a more robust analysis of how microgravity affects cellular and molecular pathways. The primary objective of this research is to analyze transcriptomics data from human cell lines exposed to microgravity to identify microgravity’s effects on cellular processes. Specifically, this study focuses on DLD-1 (solid tumor cells), MOLT-4 (hematological tumor cells), Jurkat T lymphocytic, and myelomonocytic U937 cells. DLD-1 and MOLT-4 were exposed to simulated microgravity, while Jurkat T cells and U937 cells were subjected to real microgravity during sounding rocket and parabolic flights.

This research investigates the effects of microgravity on gene expression and explores potential therapeutic interventions for damage caused by spaceflight. The study focuses on identifying which genes are differentially expressed in response to microgravity, determining which biological pathways are significantly affected and assessing whether drugs can be identified to reverse these gene expression changes.

Using a bioinformatics workflow, the research began by gathering data from NASA’s GeneLab repository, specifically targeting datasets with microgravity conditions. Differential expression analysis identified significantly altered genes, and Venn diagram analysis uncovered common DEGs across the datasets. Gene enrichment analysis provided insights into the biological significance of these genes, while drug repurposing analysis proposed potential therapeutic candidates. Finally, gene target enrichment and pathway comparisons highlighted shared biological processes between DEGs and drug gene targets, bridging spaceflight-induced gene expression changes with possible pharmacological interventions.

## 2. Results

### 2.1. Identification of Differentially Expressed Genes

**Figure 1:**
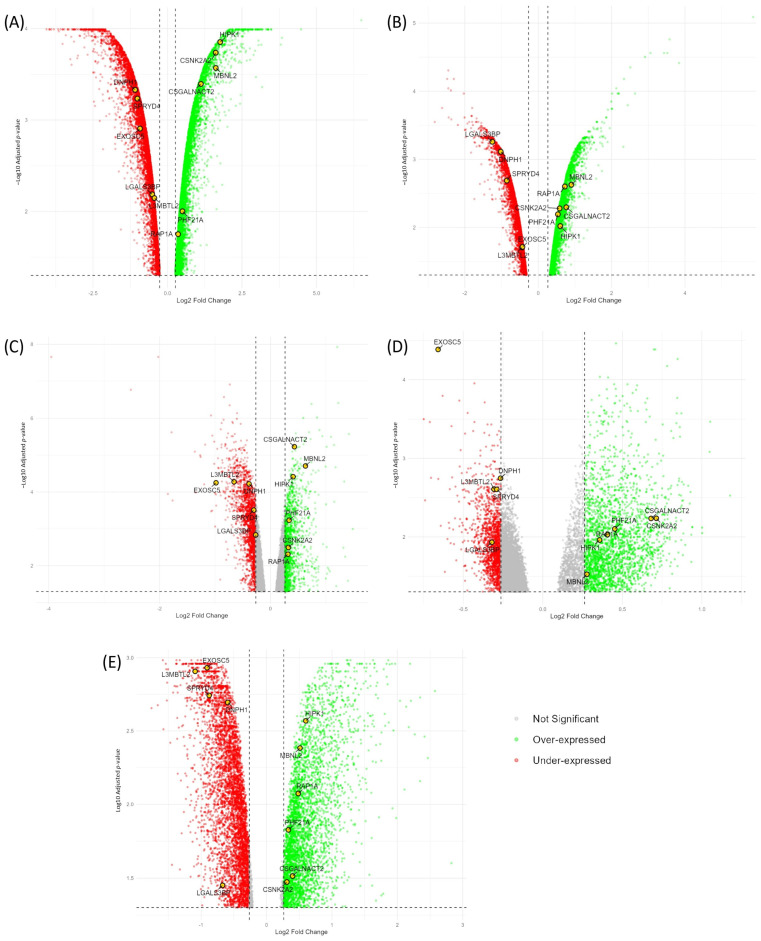
In each plot, the *x*-axis displays the log_2_FC, representing the magnitude of gene expression changes, while the *y*-axis shows the −log_10_ *q-*value, reflecting the statistical significance of these changes. The plots use color coding to differentiate between over-expressed (green), under-expressed (red), and commonly affected genes across the datasets (highlighted in yellow). (**A**) OSD-125 D, (**B**) OSD-125 M, (**C**) OSD-172, (**D**) OSD-188, and (**E**) OSD-297.

Differential expression analysis was conducted on four datasets (OSD-125, OSD-172, OSD-188, and OSD-297) with five distinct comparisons using the GEO2R tool, identifying a total of 31,744 DEGs with a log_2_FC threshold of ±0.263 and *q-*value < 0.05 (Table 1), (the full list of DEGs is in Supplementary S1). In the OSD-125 dataset, the DLD-1 and MOLT-4 cells exposed to simulated microgravity showed 10,651 and 6128 DEGs, respectively. Volcano plots (Figure 1A,B) for these cell lines demonstrate a balanced distribution of over- and under-expressed genes, though with significant outliers. Jurkat T cells in the OSD-172 dataset (20 s of microgravity) had 3017 DEGs, which were predominantly under-expressed. The OSD-188 dataset (5 min of microgravity) revealed 3756 DEGs, with an increase in over-expressed genes compared to OSD-172, suggesting longer exposure may activate more genes. The volcano plots (Figure 1C,D) show the predominance of under-expressed genes for OSD-172, and OSD-188 shows a significant increase in over-expressed genes compared to OSD-172 but fewer under-expressed genes. In the OSD-297 dataset, U937 cells exposed to microgravity with the inhibitor SKF96365 exhibited 8192 DEGs, with a slight skew towards downregulation, indicating the inhibitor’s role in amplifying microgravity’s suppressive effects (Figure 1E).

Overall, the variation in DEG numbers across datasets indicates differential cellular responses to microgravity, dependent on cell type and experimental conditions. Notably, the DLD-1 cell line in OSD-125 exhibited the largest number of DEGs, indicating a substantial transcriptional response to prolonged simulated microgravity. Eleven commonly affected DEGs across datasets are highlighted (in yellow), with their significance to be explored in the next section.

A Venn diagram analysis using Venny [20] was conducted to identify genes consistently affected by microgravity across different experimental conditions (cell type, duration, and type of microgravity). This revealed five under-expressed genes (*DNPH1*, *EXOSC5*, *L3MBTL2*, *LGALS3BP*, *SPRYD4*) and six over-expressed genes (*CSGALNACT2*, *CSNK2A2*, *HIPK1*, *MBNL2*, *PHF21A*, *RAP1A*) that were consistently differentially expressed across all the datasets. Table 2 below provides an overview of the dysregulated genes and their Entrez IDs, gene symbols, and main functions.

**Figure 2:**
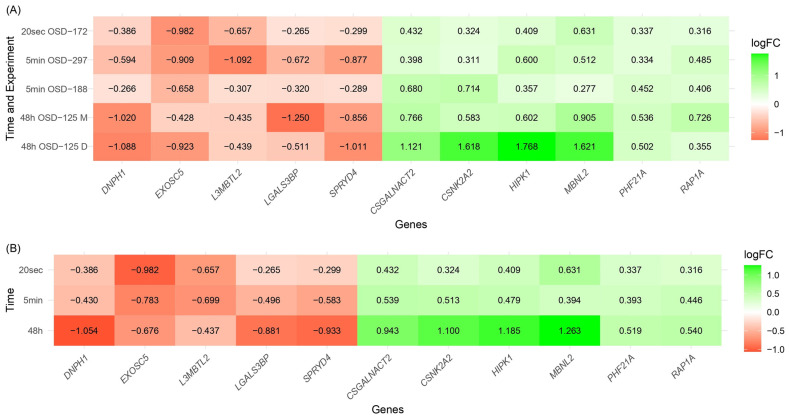
Heatmap with log_2_FC values for the 11 genes significantly affected by microgravity across different exposure durations: 20 s (OSD-172), 5 min (OSD-188 and OSD-297), and 48 h (OSD-125 D and OSD-125 M). The heatmap is color-coded, with red representing downregulation and green indicating upregulation, allowing for a visual comparison of gene expression changes over time. These findings highlight both consistent trends and variability in gene expression in response to microgravity. (**A**) All comparisons listed and (**B**) comparisons grouped by duration.

To delve deeper into the expression patterns of the 11 genes consistently affected by microgravity, we analyzed their log_2_FC values across different experimental conditions, as presented in Figure 2A. The heatmap reveals how each gene’s regulation is influenced by the duration of microgravity exposure, with notable patterns emerging for both over-expressed and under-expressed genes. To better understand the impact of microgravity exposure duration on gene expression, we calculated the average log_2_FC values for each gene across the grouped experiments: 20 s (OSD-172), 5 min (OSD-188 and OSD-297), and 48 h (OSD-125 D and OSD-125 M), and presented them in Figure 2B.

The analysis revealed consistent trends for several genes: the under-expressed genes *DNPH1*, *LGALS3BP*, and *SPRYD4* showed increasing downregulation with longer exposure to microgravity, while the over-expressed genes *CSGALNACT2*, *CSNK2A2*, and *HIPK1* showed increasing upregulation. However, some genes, like *EXOSC5* and *L3MBTL2*, exhibited weakening downregulation over time, while *PHF21A* and *RAP1A* showed less consistent upregulation. *MBNL2* displayed an unusual pattern, with fluctuating expression levels across different durations. These findings highlight both consistent trends and variability in gene expression responses to microgravity.

### 2.2. Enriched Biological Terms in Microgravity

The enrichment analysis of DEGs was conducted using GO-BP, -CC, and -MF, as well as pathway analyses via the KEGG and Reactome databases. Significant pathways were identified with a *p*-value threshold of <0.05. The number of identified pathways are summarized in Table 3, and the full list of pathways is in Supplementary S2. To explore the biological impact of microgravity, each dataset was analyzed in three forms: combined DEGs, under-expressed DEGs, and over-expressed DEGs.

Key pathways identified in the enrichment analysis provide insights into the biological processes affected by microgravity. For under-expressed genes, significant pathways include RNA processing and nucleotide metabolism, such as the nucleotide catabolic process, polyadenylation-dependent snoRNA 3′-end processing, U4 snRNA 3′-end processing, nuclear mRNA surveillance, and DNA deamination.

**Figure 3:**
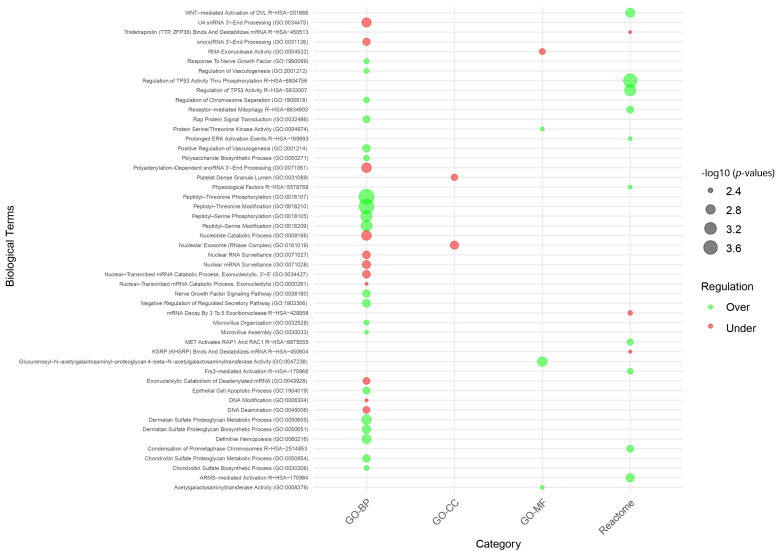
Bubble plot that visualizes the top 50 pathways identified in the enrichment analysis of DEGs under spaceflight conditions. Pathways are categorized based on their regulatory status (over-expressed in green, under-expressed in red). The size of each bubble corresponds to the −log_10_ (*p*-value), representing the significance of the pathway, with larger bubbles indicating higher significance. Pathways are grouped under different GO categories, including the GO-BP, GO-CC, GO-MF, and Reactome pathways, while KEGG pathways were excluded as they did not meet the criteria.

For over-expressed genes, pathways related to signaling and cellular stress responses were prominent, including peptidyl–threonine and peptidyl–serine phosphorylation, regulation of TP53 activity through phosphorylation, dermatan sulfate proteoglycan metabolic process, WNT-mediated activation of DVL, nerve growth factor signaling, and positive regulation of vasculogenesis. Figure 3 illustrates these significant pathways, showing the impact of microgravity on RNA processing and signaling pathways, with varying levels of gene expression indicated by bubble size and color.

### 2.3. Drug Repurposing Results

In silico drug repurposing using the L1000 DBG [21] tool identified several drugs as potential modulators of DEGs in the L1000 dataset, considering only drugs with a *q-*value < 0.05. The genes *DNPH1*, *L3MBTL2*, and *SPRYD4* had no potential candidates. Potential drug candidates and their gene targets are listed in Table 4, and a full list of modulating drugs is in Supplementary S3. Analysis of the most frequent drugs revealed trichostatin-A, CGP-60474, alvocidib, daunorubicin, fludarabine, alvespimycin, vorinostat, and parthenolide. Parthenolide had no gene targets in the Broad Institute’s database [22].

The Table 5 below illustrates the relationship between the most frequently occurring DEGs and the common drugs identified in the L1000 DGB system. The top row lists the DEGs that were most frequent across all datasets. The first column lists the most common drugs identified through the in silico drug repurposing analysis. Entries in Table 5 are marked with a “+” to indicate the correspondence between a specific drug and a DEG, suggesting that the drug affects the expression of that gene.

The enrichment analysis of the gene targets of the identified drugs across the five databases (GO-BP, GO-CC, GO-MF, KEGG, Reactome) indicated significant pathway associations for each drug (*p*-value < 0.05), which are summarized in Table 6; a full list of pathways is in Supplementary S4. Noteworthy drugs include daunorubicin, fludarabine, and vorinostat, all of which are approved or marketed, as detailed in Table 6, according to DrugBank (https://go.drugbank.com) [23] and AdisInsight (https://adisinsight.springer.com) [24].

Daunorubicin is a DNA intercalator and topoisomerase II inhibitor, fludarabine is a purine analog and DNA synthesis inhibitor, and vorinostat is a histone deacetylase (HDAC) inhibitor. Other drugs, such as CGP-60474 and Alvocidib, are cell cycle modulators, while trichostatin-A and alvespimycin affect chromatin structure and protein stability, respectively. Parthenolide showed no significant pathway enrichment due to no indicated gene targets.

### 2.4. Integration of Findings

**Figure 4:**
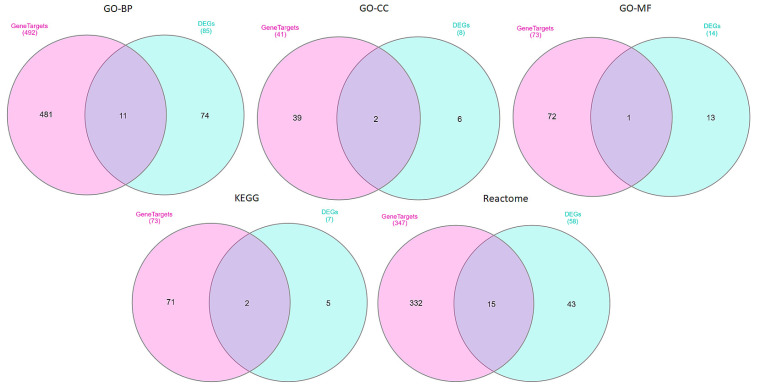
Five Venn diagrams that represent the overlap between pathways derived from DEGs and those enriched in drug gene targets across the five different databases: GO-BP, GO-CC, GO-MF, KEGG, and Reactome pathways. Each Venn diagram quantifies the number of unique and shared pathways between DEGs and drug targets, with overlaps indicating pathways common to both. The percentage in each section represents the proportion of pathways in each group.

This section analyzes overlapping pathways between DEGs and drug gene targets using the GO-BP, GO-CC, GO-MF, KEGG, and Reactome databases, focusing on statistically significant pathways (*p*-value < 0.05). Venn diagrams (Figure 4) were created to visualize these overlaps. The diagrams show common pathways, highlighting intersections that may be crucial in the biological processes or mechanisms targeted by these drugs.

Table 7 summarizes common pathways between common DEGs and drug gene targets. The pathways shared between the identified drugs and the DEGs highlight critical biological processes that are both impacted by microgravity and targeted by the drugs, making them particularly significant for therapeutic intervention. For instance, pathways such as transcriptional regulation, TP53 activity, and phosphorylation, which are disrupted under space conditions, are also modulated by drugs like vorinostat, fludarabine, and daunorubicin.

This overlap underscores the potential for these drugs to mitigate microgravity-induced gene expression changes by targeting the same dysregulated pathways, and provides a promising avenue for developing pharmacological countermeasures specifically tailored to the unique challenges posed by space environments.

## 3. Discussion

This study investigated the impact of microgravity on gene expression in human cell lines, revealing extensive changes in gene expression and biological pathways across five comparisons from various transcriptomic datasets. The cells analyzed varied in type, exposure duration, and the microgravity conditions they experienced, a diversity intentionally chosen to capture a broader spectrum of cellular responses and enhance the generalizability of our findings.

As previously mentioned in the results, simulated microgravity induced a balanced response in the DLD-1 and MOLT-4 cells, while real microgravity exposure revealed the increasing activation of gene expression over time in Jurkat cells and significant downregulation in U937 cells treated with SKF96365. Similar responses have been reported in previous studies, such as the work by Vidyasekar et al. [25], where microgravity was shown to influence cancer cell behavior through alterations in gene expression profiles. In contrast, Jurkat T lymphocytic cells exposed to real microgravity demonstrated a substantial number of differentially expressed genes (DEGs) that increased with longer exposure times. This increase in gene expression changes over time suggests the stronger activation of transcriptional processes under prolonged microgravity. Similar findings were reported in Cora S. Thiel et al.’s study [26], where T cells exhibited rapid transcriptional changes under altered gravity conditions, highlighting a time-dependent transcriptional response induced by microgravity.

This study identified 11 DEGs that were consistently affected across the conditions tested. Among the under-expressed genes, *DNPH1* plays a critical role in nucleotide metabolic processes required for DNA replication, repair, RNA synthesis, and cellular energy homeostasis, all of which are essential for cell division and the balance between cell growth and death, with implications for cancer aggressiveness and metastasis [25]. Similarly, *EXOSC5*, which is involved in RNA degradation and processing, is essential for RNA quality control and immune responses. Microgravity exposure has been shown to disrupt RNA polymerase II binding to DNA, affecting gene transcription and potentially contributing to immune dysfunction [27]. Furthermore, *L3MBTL2*, involved in chromatin modification and DNA damage repair, was also downregulated in our study, which is consistent with literature showing increased sensitivity to ionizing radiation in its absence [28,29]. Moreover, the downregulation of *LGALS3BP*, which plays a role in the immune response and cell adhesion, aligns with existing studies demonstrating that spaceflight weakens immunity [8,17,30,31]. It has been also reported that the loss of cell adhesion in various tissues results in adverse effects on cardiovascular health, immune function, bone density, and muscle strength, while potentially enhancing cancer cell apoptosis and contributing to the progression of certain forms of metastasis [32].

The upregulation of *CSNK2A2* and *HIPK1* suggests increased apoptotic signaling, p53 regulation, and protein phosphorylation. Microgravity accelerates T cell apoptosis, weakening immune function and increasing infection risks for astronauts [33]. The heightened oxidative stress in microgravity worsens cellular damage, highlighting the need for immune protection during space missions [34]. *HIPK1* also plays a role in the cellular stress response, reducing TNF-α and inflammation, suggesting protection against inflammation and metabolic disturbances [35]. Upregulation of *CSGALNACT2* has been linked to reduced inflammation, possibly related to microgravity-induced immune suppression [36]. *PHF21A*, involved in histone deacetylation, affects gene expression, cellular stress responses, and can contribute to neuroinflammation, impaired memory, and tumor formation [37,38,39,40]. *RAP1A*, involved in signal transduction and MAPK pathways, is linked to oncogenesis, and its activation increases under microgravity [41,42]. *MBNL2* regulates RNA splicing and shows variable responses to microgravity exposure. This variability suggests that prolonged microgravity exacerbates gene dysregulation, though genes like *EXOSC5*, *L3MBTL2*, and *MBNL2* showed no expected increase in dysregulation. This may indicate that RNA processing and gene regulation are better adapted to microgravity [13,43,44]. Cora S. Thiel hypothesized that gravitational forces influence chromatin structure, maintaining gene expression homeostasis [44]. A summary of the functions and relevance to space biology of the 11 common DEGs is presented in Table 8.

As noted earlier, there is limited literature on the DEGs identified in this study in relation to microgravity. One exception is *DNPH1*, which is under-expressed in Jurkat T lymphocytes and linked to cellular proliferation and transformation as a putative oncogene involved in c-Myc-mediated transformation [45]. Many microgravity-affected DEGs are related to cancer. For instance, *PHF21A*, over-expressed in our study, is implicated in colorectal cancer, enhancing lymphangiogenesis and immune cell infiltration associated with metastasis [46]. *PHF21A* disruption affects proliferation and lineage commitment, leading to disrupted cell renewal [47]. *RAP1A*, also over-expressed, is linked to esophageal squamous cell carcinoma, enhancing metastasis through increased cell migration and invasion [48]. *EXOSC5*, recognized as a tumor suppressor, has been downregulated in various cancers, including renal cell carcinoma, and plays a critical role in signaling pathways like JAK-STAT, MAPK, and WNT, which are crucial for cancer progression [49]. *LGALS3BP* and *CSNK2A2* are associated with the NF-κB pathway, which is crucial in tumor progression. *LGALS3BP* is suspected to be a tumor suppressor due to its under-expression in prostate cancer, while *CSNK2A2* over-expression promotes tumor progression, particularly in hepatocellular carcinoma [50]. *MBNL2*, typically tumor-suppressive, promotes cancer when depleted but, in some contexts or stages of cancer, contributes to cancer development [51]; it plays a key role in cardiac fibrosis, especially in the aging heart, promoting fibroblast senescence and fibrosis [52]. Lastly, the role of under-expressed *SPRYD4* is less defined, though its over-expression in cholangiocarcinoma leads to cell cycle arrest and apoptosis, while knockdown of this gene causes the opposite effect, suggesting a role in cell cycle regulation and apoptosis [53]. This complex relationship between microgravity-induced gene expression changes and cancer pathways highlights potential implications for astronaut health. Understanding these pathways could help develop countermeasures for long-term space missions.

In this study, in silico drug repurposing identified daunorubicin, vorinostat, and fludarabine as potential modulators for DEGs. Daunorubicin, a chemotherapeutic, intercalates DNA and inhibits topoisomerase II, disrupting DNA replication and transcription [54]. However, its use in space presents significant challenges. While initial studies suggested daunorubicin as a candidate for mitigating muscle atrophy in space [55], further research highlighted its potential risks. Microgravity has been shown to alter the drug’s efficacy, leading to increased migration of cancer cells; as well as being a potent chemotherapeutic agent, it possesses significant side effects, including cardiotoxicity, myelosuppression, hair loss, nausea, and vomiting [56]. This paradoxical effect underscores the need for comprehensive space-based drug testing and potential adjustments in drug dosing regimens to ensure its safety and effectiveness for astronauts. Vorinostat, an HDAC inhibitor, modifies chromatin and gene expression [57]. Its mechanism involves histone acetylation changes, impacting cancer-related pathways such as those regulated by *EXOSC5* [58]. Moreover, vorinostat’s dual role as a radioprotector and radiosensitizer [39] suggests an application for managing radiation and cancer in space, particularly with microgravity-induced epigenetic changes [59]. Fludarabine has been shown to have synergistic effects when combined with other cancer therapeutics, including HDAC inhibitors [39]. This synergy could be particularly valuable in the context of space travel, where the disruption of cellular pathways by microgravity mirrors some aspects of cancer pathology [60]. The potential for fludarabine to enhance the efficacy of other treatments, such as vorinostat, makes it a candidate worth further exploration for spaceflight applications.

These drugs, though designed for cancer, may overlap with pathways altered by spaceflight, like immune function and cellular repair. Their interaction with space-induced cellular changes highlights the need for further in vitro and in vivo testing under microgravity to ensure their safety and efficacy for astronauts.

This study’s limitations include reliance on specific cell lines, short-duration microgravity exposure, and simulated conditions that may not fully replicate long-term spaceflight effects. Differences between simulated and real microgravity could cause discrepancies in gene expression results. Gene expression is context-dependent, influenced by factors like cell type and exposure duration. Dysregulated gene effects were not validated experimentally and, while in silico analysis suggested potential drugs to combat these effects, their safety in space, particularly with radiation exposure and altered immune function, remains uncertain.

## 4. Methods

**Figure 5:**
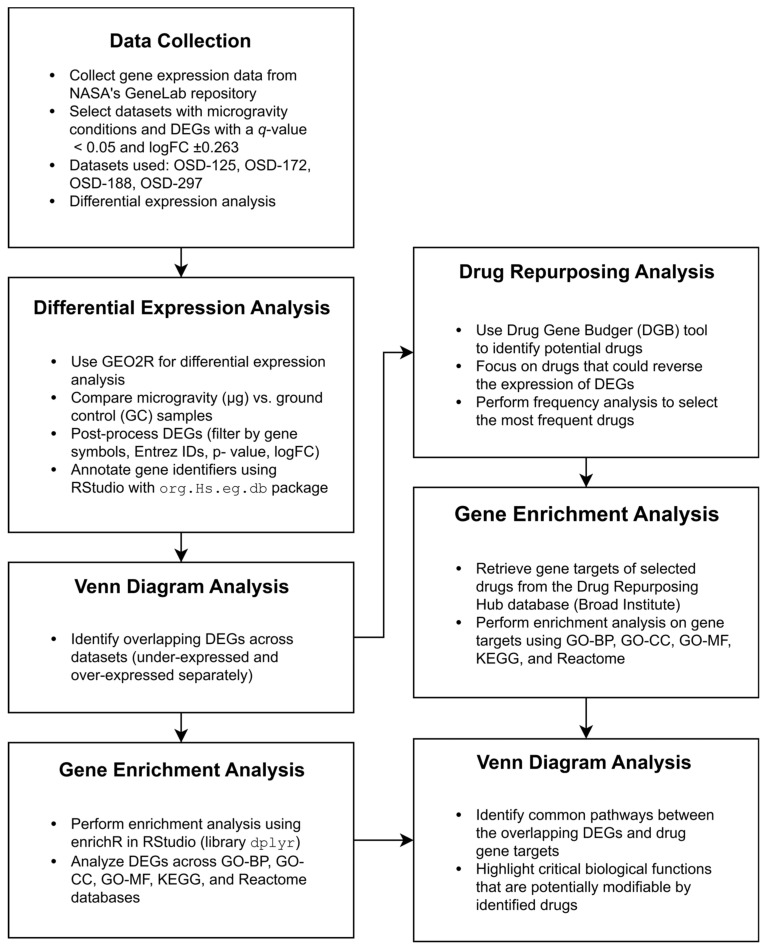
Overview of the workflow: The process includes the collection and analysis of gene expression data to identify DEGs in microgravity versus ground control conditions. This is followed by a drug repurposing analysis that identifies potential drugs to modulate these DEGs. The workflow also includes gene enrichment and Venn diagram analyses to identify overlapping DEGs and shared pathways, highlighting critical biological processes that could potentially be targeted by the identified drugs.

The workflow of the proposed methodology is described in the following diagram (Figure 5). Figure 5 depicts a comprehensive bioinformatics workflow developed for analyzing gene expression data related to spaceflight conditions. This workflow integrates various steps, beginning with the collection of gene expression data from NASA’s GeneLab repository, focusing on DEGs between microgravity (μg) and ground control (GC) conditions. Subsequent steps involve differential expression analysis, drug repurposing analysis to identify potential drugs capable of modulating these DEGs, and gene enrichment analysis to examine the biological significance of the identified genes. Venn diagram analyses help identify overlapping DEGs and common pathways, which reveal key biological processes amenable to pharmacological intervention in spaceflight contexts.

### 4.1. Differential Expression Analysis and Pre-Processing

Differential expression analysis was performed using GEO2R, a user-friendly tool from NCBI’s GEO [61]. For each dataset, microgravity samples were analyzed in comparison to ground control (GC) samples using GEO2R, ensuring proper data normalization and log_2_ transformation. DEGs were filtered to exclude duplicates, unannotated genes, and those with a *q-*value ≥ 0.05. A log_2_ fold change (log_2_FC) threshold of ±0.263 was used for biological significance. Gene identifiers were standardized using the org.Hs.eg.db package in RStudio [62] (R version 4.3.1 (2023-06-16 ucrt)). These DEGs provided the basis for downstream analyses on microgravity’s influence on gene expression.

### 4.2. Enrichment Analysis

Enrichment analysis was conducted using the enrichR package(4.3.1 (2023-06-16 ucrt)), leveraging five enrichment databases [63]. This process included annotating gene lists, assessing biological significance, and correcting for multiple hypotheses. Each gene dataset was examined in three forms: combined DEGs, under-expressed DEGs, and over-expressed DEGs. Gene ontology (GO) categories and pathway databases were employed to annotate the identified gene lists and assess their biological significance in response to microgravity. Specifically, GO-BP (biological processes) was utilized to analyze the processes influenced by differentially expressed genes, GO-CC (cellular components) provided insights into the cellular locations associated with these genes, and GO-MF (molecular functions) explored their biochemical activities [64]. Additionally, pathway analyses using the KEGG and Reactome databases were conducted to identify and characterize the molecular networks and signaling pathways significantly impacted by microgravity conditions [65,66].

### 4.3. Drug Repurposing Analysis

The in silico drug repurposing analysis was conducted using the computational tool called Drug Gene Budger (DGB) (https://maayanlab.cloud/DGB/) [21] (accessed on 7 August 2024). DGB is an online platform that uses an extensive database of drug-induced gene expression profiles to rank small molecules based on their ability to influence the expression of specified target genes. DGB offers metrics for each molecule, such as log_2_FC, *p*-value, and *q-*value, which assess the magnitude and statistical significance of the observed expression changes. The data in DGB is derived from the LINCS L1000 dataset [67], the Connectivity Map (CMap) [68], and the Gene Expression Omnibus (GEO) [69]. In our analysis, we focused on small molecules with a *q-*value < 0.05, which effectively reversed the expression of DEGs according to the L1000 dataset.

Gene targets and chemical information of the most frequent drugs were retrieved from the Drug Repurposing Hub (https://repo-hub.broadinstitute.org/repurposing) [22] (accessed on 10 August 2024) and subjected to enrichment analysis using the same five databases (GO-BP, GO-CC, GO-MF, KEGG, and Reactome). Only pathways with a *p*-value < 0.05 were kept, and the pathways from individual drugs were consolidated, removing duplicates to end up with five lists of unique pathways (one for each database) enriched across the gene targets of the selected drugs.

## 5. Data Collection

Gene expression data were obtained from NASA’s GeneLab database, a public space-related omics database [70]. Only transcriptomics datasets with microgravity conditions were included, requiring a differential expression analysis that compared microgravity-exposed samples to ground control samples, with DEGs defined as having a *q-*value of less than 0.05. Four datasets and five comparisons were chosen for further analysis: OSD-125 (DLD-1 and MOLT-4), OSD-172, OSD-188, and OSD-297. A summary of these datasets, including cell type, microgravity type, duration, and GEO access codes, is available in Table 9.

The selection of cell lines and datasets was primarily guided by the availability of transcriptomic data in the NASA GeneLab repository. Due to the limited number of publicly available datasets meeting the criteria for differential expression analysis under microgravity conditions, all suitable data were included in this study. This approach ensured the comprehensive utilization of existing resources while enabling the identification of consistently dysregulated genes across diverse experimental conditions. The variability in microgravity conditions, ranging from simulated environments (HARV bioreactors) to real microgravity during parabolic flights and sounding rockets, provides a broader context for assessing microgravity’s impact on gene expression.

## 6. Conclusions

In conclusion, our study provides a novel approach to addressing the challenges of space missions through drug repurposing. We identified several genes and associated pathways that were dysregulated in response to microgravity, highlighting potential targets for therapeutic interventions. Notably, our in silico analysis identified daunorubicin, vorinostat, and fludarabine as potential candidates for mitigating microgravity-induced effects, although their application in spaceflight conditions requires careful validation.

To our knowledge, this is the first study attempting to systematically repurpose cancer therapeutics for humans in spaceflight applications, leveraging their effects on cellular processes that are altered in microgravity. Additionally, we identified 11 consistently dysregulated genes across various experimental conditions, providing insights into the molecular impacts of microgravity on gene expression and cellular functions. Future research should focus on long-term exposure studies and validate the functional implications of these genes and drugs under actual spaceflight conditions. Expanding the range of biological systems and incorporating advanced techniques will further enhance our understanding and pave the way for effective countermeasures in space missions.

## Figures and Tables

**Table 1:** Summary of DEGs across all datasets and cell lines.

Exp. Name	Combined	Under-Expressed	Over-Expressed
OSD-125 D	10,651	5222	5429
OSD-125 M	6128	3144	2984
OSD-172	3017	1765	1252
OSD-188	3756	1419	2337
OSD-297	8192	4533	3659

**Table 2:** The gene symbols and Entrez IDs of commonly affected genes and their gene functions.

**Under-Expressed DEGs**		
**Entrez ID**	**Gene Symbol**	**Gene Function(s)**
10591	*DNPH1*	Catabolism of deoxynucleotides
56915	*EXOSC5*	RNA processing and quality control
83746	*L3MBTL2*	Regulation of gene expression, chromatin organization
3959	*LGALS3BP*	Immune response, cell adhesion
283377	*SPRYD4*	Undefined role, potential cell cycle regulation
**Over-Expressed DEGs**		
**Entrez ID**	**Gene Symbol**	**Gene Function(s)**
55454	*CSGALNACT2*	Biosynthesis of chondroitin sulfate, inflammation response
1459	*CSNK2A2*	Cell cycle regulation, DNA repair, apoptosis, circadian rhythms
204851	*HIPK1*	Apoptosis, development, stress response pathways
10150	*MBNL2*	RNA-binding protein, alternative splicing regulation
51317	*PHF21A*	Transcriptional repression, chromatin remodeling
5906	*RAP1A*	Cell adhesion, proliferation, differentiation

**Table 3:** Summary of enrichment analysis results across GO categories and pathway databases.

	Pathways of Combined DEGs	Pathways of Under-Expressed DEGs	Pathways of Over-Expressed DEGs
GO-BP	85	33	79
GO-CC	8	6	4
GO-MF	14	9	7
KEGG	7	1	17
Reactome	58	21	56

**Table 4:** List of potential drug candidates for modulating DEGs with PubChem IDs, SMILES, and gene targets.

Name	SMILES	PubChem ID	Gene Targets
Trichostatin-A	C[C@H](\C=C(/C)\C=C\C(=O)NO)C(=O)c1ccc(cc1)N(C)C	444732	HDAC1, HDAC10, HDAC2, HDAC3, HDAC4, HDAC5, HDAC6, HDAC7, HDAC8, HDAC9
CGP-60474	OCCCNc1cc(ccn1)-c1ccnc(Nc2cccc(Cl)c2)n1	644215	CDK1, CDK2
Alvocidib	CN1CC[C@@H]([C@H](O)C1)c1c(O)cc(O)c2c1oc(cc2=O)-c1ccccc1Cl	5287969	CDK1, CDK2, CDK4, CDK5, CDK6, CDK7, CDK8, CDK9, EGFR, PYGM
Daunorubicin	COc1cccc2C(=O)c3c(O)c4C[C@](O)(C[C@H](O[C@H]5C[C@H](N)[C@H](O)[C@H](C)O5)c4c(O)c3C(=O)c12)C(C)=O	30323	TOP2A, TOP2B
Fludarabine	Nc1nc(F)nc2n(cnc12)[C@@H]1O[C@H](CO)[C@@H](O)[C@@H]1O	657237	ADA, DCK, POLA1, RRM1, RRM2
Alvespimycin	CO[C@H]1C[C@H](C)CC2=C(NCCN(C)C)C(=O)C=C(NC(=O)\C(C)=C\C=C/[C@H](OC)[C@@H](OC(N)=O)\C(C)=C\[C@H](C)[C@H]1O)C2=O |c:7,25,t:17,23,36|	59996481	HSP90AA1
Vorinostat	ONC(=O)CCCCCCC(=O)Nc1ccccc1	5311	HDAC1, HDAC10, HDAC11, HDAC2, HDAC3, HDAC5, HDAC6, HDAC8, HDAC9
Parthenolide	C\C1=C/CC[C@@]2(C)O[C@H]2[C@H]2OC(=O)C(=C)[C@@H]2CC1 |c:1|	6473881	none

**Table 5:** Correspondence of the identified drugs with common DEGs in the L1000 database.

**Under-Expressed DEGs**						
	*DNPH1*	*EXOSC5*	*L3MBTL2*	*LGALS3BP*	*SPRYD4*	
Trichostatin-A		+		+		
CGP-60474		+		+		
Alvocidib		+		+		
Daunorubicin		+		+		
Fludarabine		+		+		
Alvespimycin				+		
Vorinostat				+		
Parthenolide				+		
**Over-Expressed DEGs**						
	*CSGALNACT2*	*CSNK2A2*	*HIPK1*	*MBNL2*	*PHF21A*	*RAP1A*
Trichostatin-A	+		+	+	+	
CGP-60474			+	+	+	
Alvocidib			+	+	+	
Daunorubicin	+		+	+		
Fludarabine	+		+	+		
Alvespimycin	+	+	+	+		
Vorinostat	+		+	+	+	
Parthenolide		+	+	+		+

**Table 6:** The number of enriched pathways for each drug across the different databases, their modes of action, their status, and the status of their clinical trials.

Drug Name	Trichostatin-A	CGP-60474	Alvocidib	Daunorubicin	Fludarabine	Alvespimycin	Vorinostat	Parthenolide
**GO-BP**	187	64	223	20	42	60	173	0
**GO-CC**	10	7	15	5	0	11	10	0
**GO-MF**	33	7	21	5	8	14	25	0
**KEGG**	16	20	45	0	7	15	15	0
**Reactome**	98	109	201	8	40	87	94	0
**Mode of Action**	HDAC inhibitor	CK2 inhibitor	CDK inhibitor	DNA intercalator, Topoiso-merase II inhibitor	Purine analog, DNA synthesis inhibitor	Hsp90 inhibitor	HDAC inhibitor	NF-κB pathway inhibitor
**Status**	Being researched	In trials	In trials	Approved	Approved	Being researched	Approved	Being researched
**Num. of Clinical Trials**	93	-	65	467	2197	7	276	4

**Table 7:** Common pathways with DEGs (all, *p*-value < 0.05) vs. drugs (unique, *p*-value < 0.05).

**GO-BP**	
Regulation of Autophagy of Mitochondrion	2
Regulation of Mitochondrion Organization	2
Negative Regulation of Catabolic Process	2
Peptidyl–Serine Phosphorylation	2
Peptidyl–Serine Modification	2
Phosphorylation	2
Protein Phosphorylation	2
Peptidyl–Threonine Phosphorylation	2
Peptidyl–Threonine Modification	2
Positive Regulation of Growth	1
Regulation of Cellular Component Biogenesis	1
**GO-CC**	
Nucleus	3
Intracellular Membrane-Bounded Organelle	3
**GO-MF**	
Protein Serine/Threonine Kinase Activity	2
**KEGG**	
Adherens junction	1
PD-L1 expression and PD-1 checkpoint pathway in cancer	1
**Reactome**	
Generic Transcription Pathway	4
RNA Polymerase II Transcription	4
Gene Expression (Transcription)	4
Regulation of TP53 Activity	4
Transcriptional Regulation by TP53	4
Selective Autophagy	3
Hemostasis	3
HDACs Deacetylate Histones	2
SUMOylation of Chromatin Organization Proteins	2
Chaperonin-mediated Protein Folding	2
Condensation of Prometaphase Chromosomes	2
Regulation of TP53 Activity Through Phosphorylation	2
Signal Transduction by L1	1
Metabolism	1
Transcriptional Regulation by E2F6	1

**Table 8:** The 11 common DEGs, their functions, and their implications under spaceflight conditions.

Gene	Function	Relevance for Spaceflight
*DNPH1*	Nucleotide metabolism essential for DNA replication, repair, RNA synthesis, and energy homeostasis.	Downregulation may restrict cell division, leading to cancer progression and reduced cellular energy, highlighting the need for countermeasures.
*EXOSC5*	RNA processing and quality control via the exosome complex.	Impaired RNA processing under microgravity can disrupt gene expression and immune function, necessitating RNA-focused therapeutic interventions.
*L3MBTL2*	Transcription regulation via chromatin modification and SUMOylation.	Reduced DNA damage repair increases sensitivity to ionizing radiation, underscoring its importance for astronaut safety in high-radiation settings.
*LGALS3BP*	Immune response and cell adhesion.	Downregulation compromises immunity, cardiovascular health, and tissue integrity, which are critical for prolonged space missions.
*SPRYD4*	Potential role in cell cycle regulation and apoptosis.	Altered expression may disrupt normal cell cycle processes, influencing tissue regeneration and cellular health.
*CSGALNACT2*	Biosynthesis of glycosaminoglycans and regulation of inflammation.	Upregulation reduces inflammation, providing a protective response against microgravity-induced immune suppression.
*CSNK2A2*	Regulation of the cell cycle, DNA repair, apoptosis, and circadian rhythms.	Upregulation increases apoptosis and oxidative stress, potentially weakening immune responses and promoting cellular damage in astronauts.
*HIPK1*	Apoptosis and cellular stress response.	Upregulation contributes to inflammation reduction but accelerates T cell apoptosis, impairing immunity during space missions.
*MBNL2*	RNA binding and alternative splicing regulation.	Exhibits variable responses, reflecting complex adaptations to microgravity, potentially affecting gene expression and cellular function.
*PHF21A*	Chromatin modification and transcriptional repression.	Upregulation may suppress necessary gene expression linked to neuroinflammation and cancer progression in microgravity conditions.
*RAP1A*	Signal transduction and cell growth.	Upregulation enhances MAPK pathway activity linked to oncogenesis and increased cell migration under microgravity.

**Table 9:** Summary of microgravity exposure conditions for selected experiments.

Exp. No	Cell Type	Microgravity Type	Microgravity Duration	GEO Access Code
OSD-125 D	DLD-1	Simulated (HARV *)	48 h	GSE69271 [71]
OSD-125 M	MOLT-4	Simulated (HARV *)	48 h	GSE69271 [71]
OSD-172	Jurkat cells	Real (parabolic flights)	20 s	GSE94253 [72]
OSD-188	Jurkat cells	Real (sounding rocket)	5 min	GSE94255 [73]
OSD-297	U937	Real (sounding rocket)	5 min	GSE101299 [74]

* HARV—high aspect ratio vessels.

## Data Availability

The original contributions presented in the study are included in the article and Supplementary Materials.

## Supplementary Materials

The supporting information can be downloaded at: https://www.mdpi.com/article/10.3390/ijms26031287/s1.
